# How Do Institutional Conflicts of Interest Between Pharmaceutical Companies and the Healthcare Sector Become Corrupt? A Case Study of Scholarship Donations Between Department of Clinical Anesthesiology, Mie University, and Ono Pharmaceutical in Japan

**DOI:** 10.3389/fpubh.2021.762637

**Published:** 2022-01-03

**Authors:** Akihiko Ozaki, Anju Murayama, Kayo Harada, Hiroaki Saito, Toyoaki Sawano, Tetsuya Tanimoto, Piotr Ozieranski

**Affiliations:** ^1^Medical Governance Research Institute, Tokyo, Japan; ^2^Department of Breast Surgery, Jyoban Hospital of Tokiwa Foundation, Iwaki, Japan; ^3^Department of Gastroenterology, Sendai Kosei Hospital, Sendai, Japan; ^4^Department of Surgery, Jyoban Hospital of Tokiwa Foundation, Iwaki, Japan; ^5^Department of Social and Policy Sciences, University of Bath, Bath, United Kingdom

**Keywords:** conflicts of interest, ethics, Japan, pharmaceutical companies, Mie University, anesthesiology

## Abstract

Institutional conflicts of interest (ICOIs) with pharmaceutical companies can bias internal operation of healthcare organizations. Naturally, a scholarship donation—which is a donation scheme unique to Japan, provided to healthcare organizations and their subunits to encourage educational and academic activities related to the development of new drugs—fall into the ICOI category. While anecdotal evidence exists that scholarship donations have been used as bribes by pharmaceutical companies, there has been little case study research that would illuminate the workings of this “gray area” mechanism. From this perspective, we offer an in-depth analysis of a recent scandal involving the Department of Clinical Anesthesiology, Mie University and Ono Pharmaceutical, where a scholarship donation was used by a pharmaceutical company to increase the prescription of one of its key drugs at a hospital department. Available evidence also suggests that a professor based within the department originally requested a scholarship donation from the company, which became an initial trigger of the scandal. We argue that by scrutinizing scholarship donations we can gain insight into problems specific to ICOIs between the pharmaceutical companies and the healthcare sector in Japan. In addition, scholarship donations can be understood as a form of “gifts” which have been found to underpin certain forms of pharmaceutical companies' promotional activities in Japan but also in other countries. We conclude by highlighting potential institutional remedies, which may alleviate ICOIs and corrupt behavior affecting the healthcare sector.

## Institutional Conflicts of Interest

Conflicts of interests (COIs) between pharmaceutical companies and the healthcare sector have long been a source of concern because of their adverse impacts on health policy, clinical practice, and research (1). Recent transparency initiatives have accelerated investigations into this issue, mainly in the US (2), Europe (2), Australia (3), and Japan (4). COIs between pharmaceutical companies and the healthcare sector can be principally categorized into individual COIs and institutional COIs (ICOIs). Unlike individual COIs, which refer to COIs between pharmaceutical companies and healthcare professionals (HCPs), ICOIs can reportedly arise when healthcare organizations (HCOs), any of their representatives, and their subunits (e.g., departments and institutes based within medical institutions), have financial relationships with pharmaceutical companies, and when organizational representatives have individual COIs with pharmaceutical companies (5). Although ICOIs have been relatively less studied compared with individual COIs (6), it has been reported that ICOIs could be at least just as damaging for the health and well-being of patients and general public by biasing internal operations of and research conducted in HCOs in ways which prioritize commercial interests of pharmaceutical companies over patients' and public health needs (5).

ICOIs have primarily been considered to-date in the context of research activities undertaken in the US. There are two plausible explanations. First, the introduction of the Bayh-Dole Act in 1980 has encouraged research collaborations between pharmaceutical companies and HCOs, thereby creating contexts where ICOIs can arise in relation to research activities (5). Second, the death of 18 year-old Jesse Gelsinger in 1999 highlighted how ICOIs could bias a healthy conduct of clinical trials and harm patients (7, 8). Gelsinger died 4 days after an infusion of novel adenovirus vector carrying a corrected gene while participating in its Phase 1 clinical trial, led by Dr. James Wilson, who presumably held a share of the biotech company Genovo, which was supposed to gain from the trial, at 28.5 to 33 million USD at that time (8). Further, it is reported that, in the trial, there was much space for improvement in securing informed consent from participants, particularly in terms of safety of the gene therapy (8). Currently, there are several federal rules related to ICOIs in the US, including the Open Payments Program involving disclosure of payments made by pharmaceutical and medical device companies to teaching hospitals (9).

## Scholarship Donations: Notable Source of Institutional Conflicts of Interest in Japan

Japan is the world's third largest market of pharmaceuticals with the annual sales of 79 billion USD in 2019 (10), only followed by United States and China, and is another country in which a research scandal relating to ICOIs triggered the establishment of regulatory frameworks to manage ICOIs between pharmaceutical companies and the healthcare sector. From the 2000s to early 2010s multiple instances of unethical or illicit marketing of the hypertension drug Valsartan (Diovan®, Novartis Pharma) triggered the development of legal and other regulations of ICOIs, as detailed in our previous article (11). Most importantly, the Clinical Trial Act of 2018 sought to improve ICOI regulation and transparency in clinical trials (12). Separately, in 2011 the Japan Pharmaceutical Manufacturers Associations (JPMA) published transparency guidelines for its member companies to help them govern COIs with the healthcare sector, including ICOIs, by way of publishing payments made by pharmaceutical companies, with the first public payment disclosure in the fiscal year of 2013 (http://www.jpma.or.jp/english/policies_guidelines/pdf/transparency_gl_intro_2018.pdf). Nevertheless, the JPMA has not created a single central database of payments covering all member companies or non-member companies observing its guidelines voluntarily, following the example of some European countries, such as the UK and Ireland (6, 13, 14). In light of this, Japanese investigative and medical non-profit organizations, including some of authors of this article, have independently created an open-access database for these payments, including ICOIs, starting from the fiscal year of 2016 (4, 15).

A key, yet thus far not fully explored, source of ICOIs involved in the above-mentioned Diovan Scandal is payments to HCOs taking the form of “scholarship donations” (*Shogaku-kifu (*奨学寄附*)* in Japanese) (4). Specifically, the five trials related to Diovan Scandal, which were conducted at the Kyoto Prefectural University of Medicine, Jikei University School of Medicine, Shiga University of Medical Science, Chiba University, and Nagoya University, were backed up by Novartis Pharma with a total of 1,130 million JPY (9.9 million USD; the exchange rate of 113.9 JPY per 1 USD as of October 30, 2021) of scholarship donations to these universities (16), alongside other logistic support, including statistical analyses.

Scholarship donations are a unique payment type which, to the best of our knowledge, does not exist outside of Japan. The JPMA transparency guidelines define them as donations provided to HCOs and their subunits to encourage educational and academic activities related to the development of new drugs (payment Subcategory B1 falling under Category B “Academic research support expenses”). Full details of the amounts of scholarship donations and their recipients are required to be disclosed annually by companies on their websites (4). Contracts between HCOs, or and their subunits (such as departments), and pharmaceutical companies typically undergo formal review at administrative offices of recipient HCOs (17). However, these contracts do not have to document specific purposes, costs, or time periods associated with scholarship donations (17). Further, not only can scholarship donations cover direct research expenses but also indirect costs, such as clerical support (17). Therefore, recipients of scholarship donations have much discretion in spending them, and, consequently, and because of this flexibility, scholarship donations have played an important role in the operations of many HCOs (17). For example, a 2012 survey conducted among 86 Japanese universities showed that scholarship donations from private entities accounted for 31.4% of their total research budgets (17). Further, according to Ozaki et al., they are ubiquitous in the Japanese medical field, with 71 JPMA members paying 22.1 billion JPY (194 million USD) in total in 2016 (4).

A flip side of the high discretion associated in spending scholarship donations by HCOs means that pharmaceutical companies are not allowed to specify how the HCOs are expected to use these donations (17). However, importantly, the Diovan Scandal suggests that some pharmaceutical companies have used scholarship donations to fund research on their own products (17). While the enactment of the Clinical Trials Act might have decreased such incidents, a critical shortcoming of the existing law is that it does not govern any uses of scholarship donations outside of research as its name implies (12). Indeed, they have been sometimes been used as kickbacks or informal gifts demonstrating gratitude to HCOs, or their subunits, for prescribing specific drugs (4). Therefore, the amount of scholarship donations has tended to reflect “contributions” of HCOs measured by the volume of prescriptions (4).

Given their potential implications for inducing reciprocity from HCOs, which prioritizes pharmaceutical companies' commercial interests, scholarship donations may be harming patients in routine clinical practice. However, empirical evidence is still lacking regarding motivations of HCOs and pharmaceutical companies to give or accept scholarship donations and roles played by HCPs and staff of pharmaceutical companies in this process. Further, no assessment has been done regarding the extent to which scholarship donations may originate in the Japanese tradition to emphasize *giri*, or reciprocity, in business and social interactions, as famously described in the anthropology classic “Chrysanthemum and the Sword” by Ruth Benedict (18). Finally, we lack clarity regarding the impact of this “gray” custom of scholarship donations on clinical practice in Japan.

Here, we provide a glimpse into this phenomenon using the case of a recent scandal in Department of Clinical Anesthesiology, Mie University and Ono Pharmaceutical in Japan. In so doing, we mainly drew on to the Report of the External Investigation Committee on the Issue of Scholarship Donations to the Faculty of Medicine, Mie University, which was published by Ono Pharmaceutical on August 6, 2021, otherwise indicated (17). We also use this case to draw broader lessons for ICOI management in Japan and beyond.

## The Role of Scholarship Donations in the Scandal Involving Department of Clinical Anesthesiology, Mie University and Ono Pharmaceutical

A recent scandal related to scholarship donations involved a full Professor (hereafter called “A”) and an Associate Professor (hereafter called “B”) at the Department of Clinical Anesthesiology, Mie University, and two employees belonging to Chubu Sales Department at Ono Pharmaceutical (hereafter called “C” and “D”: C was D's line manager). A graduated from the National Defense Medical College in Japan in 1993 and was appointed an Associate Professor at the Department of Clinical Anesthesiology, Mie University in April 2016. He was promoted to full Professor at the department in April 2018, and B was appointed as the Associate Professor at the department at the same time.

From December 2017 to March 2018, Professor A asked D to transfer 2.0 million JPY (17.6 thousand USD) of scholarship donations to his department to amplify its research budget, which directly preceded A's move there as professor since April 2018. A reportedly had been trying to bring junior doctors from his former workplace to Mie University to grow his research capacity, but he was limited in doing so due to a lack of research funds. A also informed D about his intention to increase the use of Landiolol Hydrochloride (Onoact®, Ono Pharmaceutical)—which is approved for several types of tachyarrhythmia (atrial fibrillation, atrial flutter, and sinus tachycardia) in Japan, saying “With 2.0 million JPY of scholarship donations, I'll make Onoact's sales at the university the best in the country.” D apparently understood Professor A's strong motivation to increase the use of Landiolol Hydrochloride and considered this as a business opportunity for Ono Pharmaceutical, and thus he outlined a plan to his boss, C, involving making a scholarship donation to A's department. Initially launched in Japan in 2002, Landiolol Hydrochloride was not a leading product for Ono Pharmaceutical, accounting only for 1.8% of its total sales (5 billion JPY [43.9 million USD]/280 billion JPY [2.5 billion USD]) in 2018. In light of this, C initially declined D's plan.

However, C was notified at the end of 2017 that some slots for scholarship donations, which were managed by Ono Pharmaceutical's head office, happened to be left in the fiscal year of 2017 (April 2017 to March 2018). Also, each regional sales director, including C, was instructed to identify candidates for receiving the scholarship donations to use up the budget. Thus, C confirmed with his line manager E at the company's head office that Department of Clinical Anesthesiology at Mie University could be a candidate for obtaining the remaining slots of scholarship donations. Thus, under C's instructions, D discussed with Professor A how many vials of Landiolol Hydrochloride A and his staff would be able to utilize if a scholarship donation was made following to A's department. For example, on January 23, 2017, D wrote to C, “Last night, we (A and D) improved the report's pile-up chart and had a concrete discussion. 500V (vials) is the target and he (A) says it is possible. I have been told that it is possible to reach the 500V mark.” Further, around February 2018, A and D prepared an extensive document known as a “case accumulation table” to calculate in detail how much Landiolol Hydrochloride could be prescribed at the Mie University Hospital. The document stated, for example, how many vials of Landiolol Hydrochloride would be prescribed if the drugs were used during and after a cardiac operation, how many operations could be expected, and so on. Following this, C received a response from E by February 15, 2021 at the latest, “Okay, let it go through that way.” On this basis, C thought that he had obtained the head office's approval for the contribution of scholarship donations to the Department of Clinical Anesthesiology at Mie University. By February 26, 2018, the General Affairs Department at Ono Pharmaceutical's head office approved a donation to the Department of Clinical Anesthesiology at Mie University, and on March 20, 2018, a scholarship donation of 2 million JPY (17.6 thousand USD) was transferred to a savings account in the name of Mie University.

On March 22, 2018, Professor A sent an email to the staff at the Department of Clinical Anesthesiology with the following remarks: “Ono (Pharmaceutical) has agreed to give us 2 million (JPY) in March; we want Ono (Pharmaceutical) to become our mainstay and we want to become the top (department) in Japan in terms of Onoact use; from April, in the prevention and treatment of tachycardia during extubation, I would like to increase the use of Onoact in a discreet manner (even if it is not used), with 50 mg 1 A as the basic usage; please consider this in your own cases; It's hard to talk about the above publicly. Anyway, I want to get ahead in research, so please understand my intention and do well.”

Acting on Professor A's instructions, Associate Professor B began to dissolve and prepare 50 mg vials of Landiolol Hydrochloride for use in operations other than those in which he was in charge from around April 2018, regardless of whether he used it or not. In October 2018, B further began to dissolve and prepare 150 mg vials of Landiolol Hydrochloride, many of which were thrown away without being used for concerned cases (neither C nor D were reportedly aware of the discarding taking place). To create the impression that the discarded Landiolol Hydrochloride had been used for patients, B began to make false entries in the usage history of their electronic medical records. Consequently, the annual sales of Landiolol Hydrochloride to Mie University Hospital increased rapidly from 1.1 million JPY (9.6 thousand USD) in 2017 to 2.5 million JPY (21.5 thousand USD) in 2018 and 5.0 million JPY (43.5 thousand USD) in 2019. In the first half of 2020, when the issue of problematic scholarship donations was made public, the figure had fallen sharply to 410 thousand JPY (3.6 thousand USD). Needless to say, a considerable part of the increase seems to have been accounted for by the disposal of unused portions by Associate Professor B. This suggests that, in exchange for the scholarship donation, A and B used fraudulent means to help increase sales of Landiolol Hydrochloride, which, in turn, led to an increase in the amount of Landiolol Hydrochloride prescribed and sold at Mie University Hospital.

A whistleblower divulged this scandal, and unfortunately, ten anesthesiologists resigned from the Mie University (six in September 2020 and four in October 2020) for unknown reasons and only four active anesthesiologists remained in the department (19), delivering a significant blow to local patient care. The scandal also led to the suspension of the anesthesiology residency program at the university in October 2020, and some of those who resigned highlighted the suspension as the reason of their leave (19).

Associate Professor B was arrested by the Tsu District Local Prosecutor's Office on December 3, 2020 (20), and the remaining three involved in the scheme were arrested by the same office on January 27, 2021. The trial of Associate Professor B, who had falsified medical records and made fraudulent claims, started in March 4, 2021, and was sentenced by the Tsu District Court on April 22, 2021 to two years and six months in prison and four years probation on charges of fraudulent production of public electromagnetic records, fraudulent use of public electromagnetic records, and fraud. Then, the trials of C and D started in May 14, 2021, and both fully admitted to using scholarship donations as kickbacks for increasing prescriptions at the Department of Clinical Anesthesiology, Mie University. On June 29, 2021, the Tsu District Court sentenced them equally to eight months' imprisonment and three years' probation. The trial of Professor A has not been started yet as of November 20, 2021.

Following the arrest of two employees of Ono Pharmaceutical on January 27, 2021, Mie University suspended its business relationships with Ono Pharmaceutical from January 27 to September 26, 2021 (21). Further, Ono Pharmaceutical dismissed C and D with admonition as of July 31, 2021, with other notable disciplinary actions including the demotion of the general manager of the sales division and a 3-month pay cut of 30% for the company president, who had a background in sales (22). To the best of our knowledge, E, who eventually permitted a contribution of scholarship donations to Mie University, apparently avoided a severe penalty. Reportedly, E did not remember well about this decision of himself. Consequently, in this case where the decision was made by the head office of Ono Pharmaceutical, the employees at the end of the line suffered the heaviest punishment. The external investigation committee of this incident claimed that it would be going too far to immediately dismiss the company's lack of compliance by making two arrests. The company also announced that it would discontinue the scholarship donations in 2021, and that it would consider a different method of funding for 2022 and beyond (22). The JPMA suspended the membership of Ono Pharmaceutical on September 16, 2021.

## Generalizability and Uniqueness of this Case

It is true that there may be some aspects that cannot be generalized just with this single case study, but this case clearly shows the risk of scholarship donations being used as kickbacks or briberies to increase the level of prescriptions of specific drugs within HCOs. It is also important to recognize that the professor of the department (A) and some staff of Ono Pharmaceutical cooperated and conspired in drawing the scholarship donation from the company and increasing the prescriptions and sales of drugs in return. At last, contrary to his wish to expand his department, which would potentially benefit local residents and patients in Mie Prefecture, clinical anesthesiology department at the university collapsed. While Japanese culture underlines *giri*, or reciprocity, in business and daily interactions (18), it may not be reasonable to consider scholarship donations as its example because the relationships described here are predominantly based on financial incentives of both parties rather than a deep social bond where *giri* is expected to arise (18).

Instead, we should rather interpret scholarship donations as an expression of the industry's promotional activities, including those targeting HCOs. In this respect, the “Study on Corruption in the Healthcare Sector,” published by the European Commission, offers particularly valuable insights (23). According to the study's criteria for categorizing corruption, this case, which used gifts to influence prescribing practices, can be categorized as an example of Improper Marketing Relations (Table 1) (23). While a simple generalization is difficult, like other gifts involved in unethical or illicit marketing, scholarship donations may be instrumental in establishing a “pharmaceutical gift cycle” (24). This mechanism involves “extended” or “generalized” reciprocity between donors and recipients. That is, gifts made by pharmaceutical companies do not need to be reciprocated immediately, and they do not necessarily involve a quid pro quo—put differently, those who make gifts do not necessarily state explicitly what they want to see in return. This means that they may not be straightforward bribes, but an incentive to maintain positive long-term relationships between the two sides. Notably, even small gifts may impact physician's prescribing behavior (25) or actions taken by patient organizations (26). Similar pharmaceutical gift cycles have been reported in other Asian countries such as China (27).

**Table 1:** Improper marketing relations between Department of Clinical Anesthesiology, Mie University, and Ono Pharmaceutical through scholarship donations.

Actors	Professor and Associate Professor at Department of Clinical Anesthesiology
	Employees of Ono Pharmaceutical
Subtypes	Direct prescription influencing
Features	Improper marketing relationships were created through money.
Drivers	To increase research budgets
Prevalence	Seventy-one Japanese pharmaceutical companies belonging to Japan Pharmaceutical Manufacturers Association payed 22.1 billion JPY (194 million USD, an exchange rate of 113.9 JPY per 1 USD as of October 30, 2021) of scholarship donations in total in 2016, though this is the only example that has developed into a criminal case.

What makes this case unique, however, was Professor A's motivation involving not self-enrichment but increasing the research budget of his hospital department, according to his words cited above (17). Indeed, it seems that this motivation compromised all ethical and other professional standards that he should have upheld as a medical doctor, but we can also speculate that A was responding to increasingly difficult financial circumstances that surround Japanese universities as well as the broader incentive structure in the Japanese medical milieu, and particularly in the field of anesthesiology.

In the Japanese medical field, research outputs have been regarded as a key factor in academic promotion, including securing professorial positions or being appointed an executive board member of a medical schools and/or professional associations. Importantly, the tendency to maximize research outputs, while potentially compromising rules of good scientific and medical practice, seems particularly strong among some Japanese anesthesiologists, as suggested by the relatively high prevalence of scientific misconduct in this medical specialty in Japan. For example, according to the Retraction Watch, an online platform tracking retracted scientific publications, Japanese anesthesiologists Drs. Yoshitaka Fujii and Yuhji Saitoh were respectively ranked as the first (183 publications) and seventh position (53 publications) in the number of retracted articles globally (28). Further, Dr. Hironobu Ueshima, a Japanese anesthesiologist was found guilty of fabricating data and other misconduct in 142 publications in 2021, a majority of which have not been retracted yet (29). One possible reason of the notoriety of the Japanese anesthesiology field is that anesthesiologists enjoy fewer career choices compared to other medical specialties in Japan. For example, it is common for mid-career Japanese doctors to start their own clinics, which is advantageous for work-life balance and personal finances. However, this path is typically unavailable for anesthesiologists given the nature of this specialty. Thus, competition for academic excellence may be more intense in anesthesiology than elsewhere.

It is possible that Professor A saw a higher research budget as instrumental in maximizing the quality and quantity of research outputs published by himself and his research team. Indeed, he might have sought to use an increased research budget, and the improved publication prospects, as a way of attracting young and promising anesthesiologists, according to his words cited above (17). In this context, we cannot overlook the fact that a total value of Management Expenses Grants, formal governmental supports on national universities in Japan, has dropped from 1.2 trillion JPY (10.8 billion USD) in 2004 to 1.1 trillion JPY (9.3 billion USD) in 2020 (30). Indeed, these trends have placed extra pressure on university staff in gaining research budgets, including Professor A, and have made them to pursue private funders, including pharmaceutical companies, ultimately sometimes leading to their corrupt behavior as accentuated in this case.

Another important discussion point is the interpretation of the falsified medical records and fraudulent claims made by Associate Professor B. Although it is difficult to conclusively decipher B's motivations with the available evidence, his conduct demonstrates distorted governance which prioritized following instructions given by Professor A over ethical rules that should be binding for medical doctors. In general, professors have strong discretion in shaping the operation of their departments in Japanese university hospitals, which are typically characterized by strong hierarchical structures (31). Another problem is the lack of external oversight mechanisms and thorough reporting by HCO subunits on their financial relationships with industry, a challenge also identified in collaborations between pharmaceutical companies and public hospitals in England (32).

What is similarly important to discuss is a lack of proper ethical governance in Ono Pharmaceutical. In this case, the need to spend the budget for scholarship donations was prioritized over a proper assessment of A's department. In addition, the individual who permitted this contribution at the head office did not remember his decision clearly. Further, strict penalties of dismissal were applied to the staff directly involved, while the senior staff responsible for managing them suffered only much lighter penalties. These observations suggest problems in the governance not only of scholarship donations but also wider problems in the organizational culture of the company. Indeed, similar problematic organizational culture was revealed in the scandal of Astellas Pharma in the Europe (33).

## Potential Remedies of Scholarship Donations and Broader Perspective on Institutional Conflicts of Interest

This case offers important lessons for the central government, university hospitals, and pharmaceutical companies in Japan. With regards to the central government, they should reformulate the scholarship donation scheme, originally designed to supplement a shortage of research budgets in universities just with public research grants. While the scheme achieved its original goal to some extent, insufficient consideration was given to the possibility of scholarship donations exacerbating ICOIs with pharmaceutical companies. Thus, government should propose a more transparent scheme of donations by private entities particularly with regards to university hospitals, where ICOIs would jeopardize patients' health and well-being. Further, we propose the government to consider interventions that would help university hospitals become financially more independent, such as an increase of medical fees related to treatments using cutting-edge technology, including chimeric antigen receptor T-cell therapy and robot assisted surgery.

With regards to university hospitals, governance within their subunits should be revisited so as to improve transparency. A concerning fact is that stricter regulation within university hospitals might lead to the development of spin-off organizations among their staff so that they can collect funding while avoiding hospital regulations (34). Indeed, Professor A and Associate Professor B developed the spin-off organization for that purpose and used it to accept bribery from the medical device company Nihon Kohden Corporation, leading to another criminal case (17). While we aim to cover this incident in our future work, at this stage, we can at least propose that there should be external oversight mechanisms to detect corrupt behaviors in subunits of university hospitals, including their use of spin-off organization outside of the hospitals.

With regards to pharmaceutical companies, the abolishment, or at least an overhaul of, scholarship donations is necessary. Indeed, we can see an increasing trend among pharmaceutical companies to revisit disadvantages of scholarship donations, which includes unclearness in the purpose for making the donations. For example, leading domestic pharmaceutical companies, including Takeda Pharmaceutical Company (2021) and Astellas Pharma (2019), have abolished scholarship donations. In addition, as a lesson of this case, the entire governance from pharmaceutical companies is in need of improvement. We also believe that the general public, patients, and policymakers in Japan and other countries around the world should enhance their awareness on social responsibility of pharmaceutical companies conduct. While Ono Pharmaceutical was suspended for the JPMA's membership following the incident described in this paper, the company did not experience substantial social damages such as dropped stock prices despite its questionable disciplinary actions, which reflects the apparent lack of financial impact of a recent scandal involving off-label drug promotion by Astellas Pharma in Europe (33).

Also, from a broader perspective, this case demonstrates how ICOIs can bias the internal operation of HCOs and compromise the standard of clinical practice, indicating the need for specific regulations to control its negative impact on patient care. In the United States, pharmaceutical companies only pay for laboratories under research contracts, because unrestricted donations in exchange for prescription can be considered as a bribery or kick-back (35). Further, in the United Kingdom, the industry's self-regulatory body, the Prescription Medicines Code of Practice Authority, operating under the Association of the British Pharmaceutical Industry, adopts strategies to name and shame the offending companies by publishing reports of all cases it considers on its website irrespective of the verdict (https://journals.sagepub.com/doi/pdf/10.1177/2631309X20970477). In contrast, there is no solid framework to regulate ICOIs within and/or outside of the JPMA in Japan, indicating the urgent need for a broader initiative to govern ICOIs beyond clinical trials. Incorporating regulations similar to those adopted in other leading countries would likely improve ICOI governance in Japan. Further, countermeasures placing more emphasis on loss aversion such as suspension of medical licenses in these problematic behaviors among medical doctors may be important (36). Given that the social mechanisms underpinning scholarship donations can be found in other forms of gifts involved in pharmaceutical promotion, we believe that the lessons emerging from our study would be similarly applicable to other nations that have not incorporated such legal- or self-regulation frameworks yet.

## Data Availability Statement

All the data and evidences cited in this article are publicly available. Further inquiries can be directed to the corresponding author.

## Author Contributions

AO wrote the manuscript. All the authors contributed to the conception and design of the study, critical revision of the paper, and read and approved the final manuscript.

## Conflict of Interest

AO receives personal fees from Medical Network Systems outside the scope of the submitted work. TT receives personal fees from Medical Network Systems and Bionics Co. Ltd. outside the scope of the submitted work. PO's Ph.D. student was supported by a grant from Sigma Pharmaceuticals, a UK pharmacy wholesaler and distributor (not a pharmaceutical company). The Ph.D. work funded by Sigma Pharmaceuticals is unrelated to the subject of this paper. The remaining authors declare that the research was conducted in the absence of any commercial or financial relationships that could be construed as a potential conflict of interest.

## Publisher's Note

All claims expressed in this article are solely those of the authors and do not necessarily represent those of their affiliated organizations, or those of the publisher, the editors and the reviewers. Any product that may be evaluated in this article, or claim that may be made by its manufacturer, is not guaranteed or endorsed by the publisher.
